# *In Situ* Investigation the Photolysis of the PAHs Adsorbed on Mangrove Leaf Surfaces by Synchronous Solid Surface Fluorimetry

**DOI:** 10.1371/journal.pone.0084296

**Published:** 2014-01-03

**Authors:** Ping Wang, Tun-Hua Wu, Yong Zhang

**Affiliations:** 1 School of Environmental Science and Public Health, Wenzhou Medical University, Wenzhou, China; 2 School of Information and Engineering, Wenzhou Medical University, Wenzhou, China; 3 State Key Laboratory of Marine Environmental Science (Xiamen University), Environmental Science Research Center of Xiamen University, Xiamen, China; University of Akron, United States of America

## Abstract

An established synchronous solid surface fluorimetry (S-SSF) was utilized for *in situ* study the photolysis processes of anthracene (An) and pyrene (Py) adsorbed on the leaf surfaces of *Kandelia obovata* seedlings (*Ko*) and *Aegiceras corniculata* (L.) Blanco seedlings (*Ac*). Experimental results demonstrated that the photolysis of An and Py adsorbed on the leaf surfaces of two mangrove species under the laboratory conditions, followed first-order kinetics with their photolysis rates in the order of *Ac*>*Ko*. In addition, with the same amount of substances, the photolysis rate of An adsorbed on the same mangrove leaf surfaces was much faster than the adsorbed Py. In order to investigate further, the photolysis processes of An and Py in water were also studied for comparison. And the photolysis of An and Py in water also followed first-order kinetics. Moreover, for the same initial amount, the photolysis rate of the PAH in water was faster than that adsorbed on the leaf surfaces of two mangrove species. Therefore, photochemical behaviors of PAHs were dependent not only on their molecular structures but also the physical-chemical properties of the substrates on which they are adsorbed.

## Introduction

Polycyclic aromatic hydrocarbons (PAHs) are well-known environmental persistent pollutants listed as priority pollutants by European Union and the U.S. Environmental Protection Agency due to their teratogenic, carcinogenic and mutagenic [1], [2]. The persistence of PAHs in the environment poses a potential threat to human health through bioaccumulation and biomagnifications via food chains [3]. Arising from both natural and anthropogenic sources, PAHs are ubiquitous in different natural phases such as plant, soil, sediment, water and air [4]–[6]. Thus it is of great importance to investigate the transport and transformation of PAHs in the environment. Many PAHs, especially with four or more rings, are generally recalcitrant to be biodegraded in the environment because of their low water-solubility [7]. These compounds are more likely to be affected by sedimentation and photooxidation [8]. Therefore, studies on the photolysis processes of PAHs are extremely important in order to evaluate the fate and transformation of these hazardous compounds in the environment, and have being attracted great attention [9], [10]. At present, most studies are focus on the photolysis of PAHs in liquid medium (e.g., water, ethyl alcohol and acetone) or adsorbed on solid particles (e.g., fly ash, carbon black silica gel, porous glass, clay sand and active aluminum) [11]–[13]. It is well known that vegetation plays a key role in the environmental fate of PAHs [1]. Only a few studies involve the photolytic behaviors of PAHs adsorbed on vegetation [7], [14], [15].

Mangroves are various kinds of trees growing along the coastlines of tropical and sub-tropical regions. Because of their special ecological functions, mangroves are considered to be reservoir of lipophilic contaminations, including PAHs from various sources [16]–[18]. Mangrove leaves are common covered with large surface areas and thick waxy layers, which can accumulate the lipophilic PAHs in atmosphere [18]. Ke et al have investigated the fate of PAHs contamination in a mangrove swamp after an oil spill accident. They have believed that photolysis is one of the important way to remove the PAHs adsorbed on the mangrove leaves [19]. Thus it is of great significance to study the photochemical behaviors of PAHs adsorbed on mangrove leaves. However, recent studies on the photolysis of PAHs adsorbed on plant leaves are mostly entirely destructive sample extraction, which can not realize *in situ* monitoring the photolysis of PAHs on the leaves. In addition, the amount of PAHs volatilization from the leaf surfaces into the atmosphere and entering into the inner leaf tissues could not be ignored during the long duration experiment [14], [15]. To overcome the limitations of the traditional extraction procedures, Wild and his co-workers have used the two-photon microscopy to track directly some PAHs adsorbed on the plant leaves [20], [21]. In our previous studies, the two-photon microscopy has also been utilized for visualization three typical PAHs into the inner tissues of two mangrove species [22], [23]. However it is very expensive to purchase and maintain that instrument, which could be difficult to be widely used. Optical fiber with high light focalization, low weight and small size could be used for *in situ* investigation of pollutants adsorbed on solid substrates [24]–[27].

In our previous studies, a fiber-optic fluorimetry has been used for direct investigation the photolysis of some PAHs adsorbed on the leaf surfaces of three mangrove species [7], [28]. Nevertheless, the photolysis behaviors of PAHs adsorbed on the mangrove leaf surfaces have not been fully explored. Questions without answers are still forthcoming. What are the main transformation pathways of the PAHs adsorbed on the mangrove leaf surfaces? Is photolysis significant for the fate of PAHs in the environment? Are the photolysis mechanisms of different kinds of PAHs adsorbed on mangrove leaves similar? What are the photolytic products of the PAHs? How to get the photolytic products from the mangrove leaf surfaces? These questions are of great importance for us to learn more about the environmental behaviors of the adsorbed PAHs on mangrove leaves. Moreover, a synchronous solid surface fluorimetry (S-SSF) combined with an optical fiber and a fluorescence spectrophotometer has also been established for *in situ* determination of the PAHs adsorbed on mangrove leaves in our previous work [27]. The S-SSF method has many obvious advantages: it was easy, rapid and inexpensive to purchase, maintain and operate. Additionally, the original existing forms and the distribution of the PAHs adsorbed on the mangrove leaf surfaces could be easily determined in situ, which would be impossible for traditional methods, such as GC-MS, GC, and HPLC, which need an extraction of sample before analysis. Furthermore, no complex pretreatment involving a large amount of organic solvent is needed, and it takes less than 1 minute for the determination of one sample. To further demonstrate the tremendous scope of this method, the S-SSF was also applied for *in situ* investigation the photolysis of typical PAHs adsorbed on the mangrove leaf surfaces under the laboratory experimental conditions. In this work, anthracene (An) and pyrene (Py) were selected as the model PAHs, the viviparous seedlings of *Ko* in salt-resistance and the non-viviparous seedlings of *Ac* in salt-tolerance were studied as the typical mangrove species. In addition, with the same initial amount the photolysis of An and Py in water was also studied for comparison.

## Materials and Methods

### Apparatus and compounds generation

The spectra of PAHs were obtained utilizing a Cary Eclipse (Varian, Habor City, California) equipped with a 150-W xenon flash lamp. A fiber optic accessory with solid sample tip was installed on the fluorospectrophotometer and aligned as described in the user instructions supplied with the accessory. An optical fiber was used for fluorescence measurements. The instrumental parameters were as follows: excitation and emission slits were set at 20 and 10 nm, scan speed was performed as 120 nm.min^−1^, photomultiplier tube voltage was 600 V, and an ultrasonic cleaning device (Model KQ3200, power 150W, frequency 40 kHz) was also used to extract the leaf-wax on mangrove leaf surfaces and the wax content of the two specific mangrove leaves was quantified by the method in our previous work [18], [27].

A high pressure mercury lamp equipped with the optical fiber (CHF-XM500 W, Beijing Trusttech, Co., Ltd., China) was utilized as the light source for the photolysis study of PAHs adsorbed on the leaf surfaces of two mangrove species. In order to keep the emit light intensity steady during the experiment, the light intensity on the leaf surfaces was controlled by adjusting the height between the mangrove leaf surfaces and the optical fiber probe of the mercury lamp.

The stock solution of An and Py (Aldrich, purity>99%, USA) were prepared by dissolving the solutes in acetone in the brown volumetric flask, respectively. These solutions were then ultrasonicated for 30 min to aid solubilization and stored at 4°C in darkness to avoid possible photodegradation. Working solutions of An and Py were prepared by dilution of the stock solutions in acetone before use, respectively.

### Plant preparation

**Figure 1:**
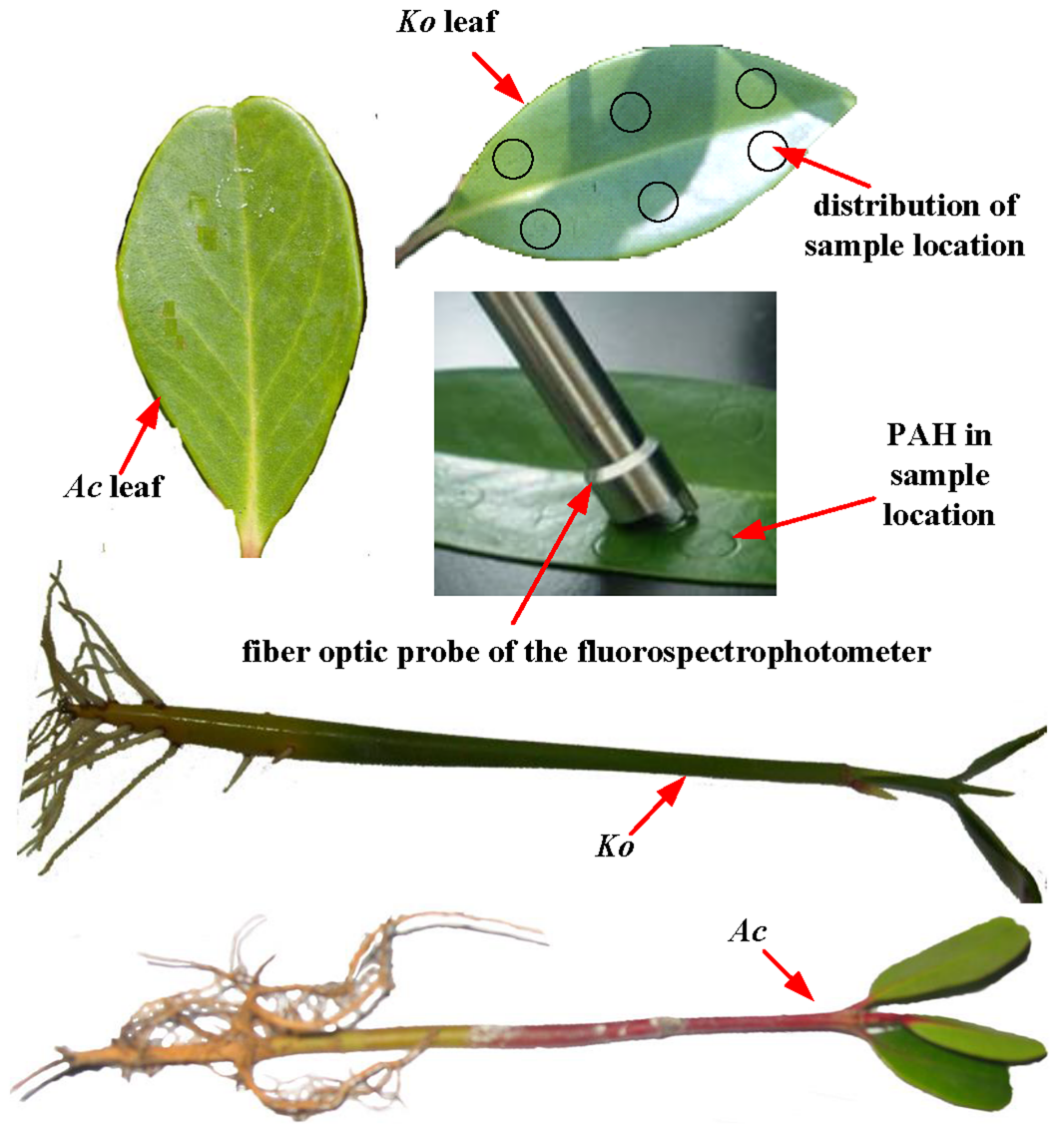
The leaves and seedlings of *Ac* and *Ko* and the distribution of sample location on leaf surface.

Mature hypocotyls of *Ko* and one-year-old *Ac*, were collected from a mangrove forest growing in Cao Putou village, Longhai city, China (longitude: 24°29′3″; latitude: 118°5′59″; altitude: 0 m above sea level). This mangrove forest is wild and does not belong to any individuals or organizations, thus no specific permissions were required for these activities. In the experiment, only a few mangrove seedlings were collected, which neither involved endangered or protected species nor vertebrate studies. The seedlings of the two mangrove species (Figure 1) with similar length and fresh weight were then planted on sand in pots partially submerged in nutrient Hoagland solution [27]. The sample preparations were all based on the methods of our previous studies [22], [23], [27]. The leaves of the two mangrove species (Figure 1) with similar length and fresh weight were collected from these mangrove seedlings cultivated for about ninety days. After the collection of the leaves, experiments were carried out as soon as possible. The picked fresh mangrove leaves were carefully rinsed with distilled water to remove surface silt. After air drying, six circles of 0.5 cm radius were drawn on the upper surfaces of each leaf with a pencil [27]. The distribution of the sample location was shown in Figure 1. The size was the same as the light circle formed by the fiber optical probe. With the use of a micropipette, a certain amount of An (44.5 ng.spot^−1^ and 445 ng.spot^−1^) and Py (50.5 ng.spot^−1^ and 505 ng.spot^−1^) acetone solutions were applied as homogeneous layers to the circles of upper leaf surfaces respectively. After evaporation of the acetone from the leaf surfaces at room temperature, a series of similar-sized spots were formed, each spot indicating a sample location (Figure 1).

### Photolysis of PAHs on the leaf surfaces of two mangrove species

The leaves of *Ko* and *Ac* with PAHs adsorbed on them were put under the mercury lamp whose light is guided by an optical fiber to avoid heating effects on the photolysis processes caused by the mercury lamp. By adjusting the height of the fiber optical probe of mercury lamp, the diameter size formed by the mercury lamp was fixed as the same as the length of the leaf blade during the course of the experiment. And the center of the light spot was directly faced the center of the leaf vein. Thus, the light intensity accepted by each sample location of the leaves is basically the same. In addition, the proper illumination intensity on the leaf surfaces was controlled at 3.53(±0.07)×10^4^ lx, which was determined by a ZDS-10 illuminometer (Shanghai, China) in the photolysis experiments. After a certain period of illumination, the leaves of *Ko* and *Ac* were then placed under the fiber optic probe of the fluorospectrophotometer, respectively. And the relative S-SSF intensities of the PAH adsorbed on the leaves were directly determined. To avoid the interference from the scattered light, an angle between the fiber optic probe of the fluorospectrophotometer and the tested mangrove leaves was kept at 45° during the experiment. Four mangrove leaves of each species were involved, yielding 24 locations and 72 measurements for each species. The value of relative S-SSF intensity presented represents the average behavior of the adsorbed PAH on different locations of each mangrove species. The mean values of the data were utilized to express the final results. And the data processing was based on the method of our previous studies. Statistical analysis for the variation of the fluorescence intensity obtained was performed using the statistics package for social science (SPSS) 13.0 for Windows. The significant differences in the results were determined using a one-sample t-test at the 95% confidence level (p<0.05 means that a remarkable difference existed) [7], [28].

### Photolysis of PAHs dissolved in water

As mentioned above, most studies are focus on the photolysis of PAHs in liquid medium (e.g., water, ethyl alcohol and acetone). The water as the homogeneous media is very simple. Thus, the mechanisms of the photolysis of PAHs in water are relatively simple and much work has been done in relevant researches. The photolytic behaviors of the PAHs adsorbed on the leaf surfaces have been found very common in the nature. And the leaf as the heterogeneous substrate is very complicated. Thus, few studies involve the photolysis processes of PAHs adsorbed on leaf surfaces. In addition, the photolysis mechanisms of the PAHs adsorbed on mangrove leaves have been hardly studied. Therefore, in order to further study the photolysis mechanisms of the PAH adsorbed on mangrove leaf surfaces, the photolysis of the same initial amount of PAH dissolved in water was also investigated. A self-made photodegradation reactor in our previous studies was used to study the photolysis processes of the PAHs dissolved in water [27]. With the use of a micropipette, a certain amount of An (44.5 ng.spot^−1^) and Py (50.5 ng.spot^−1^) aqueous solutions were added into the reactor which was directly put under the mercury lamp, respectively. After a certain period of illumination, the reactor was then put into the fluorescence spectrophotometer. And the relative fluorescence intensities of An and Py in aqueous solution were obtained, respectively. Because of the small volume of the reactor, working solutions of An and Py need not to be stirred and could realize *in situ* determination of the PAHs during the photolysis processes. These experiments were repeated three times.

### Data processing

It is reported that two important parameters (*C*_t_ and *C*_0_) should be determined in study of the photolysis processes of the PAH. The *C*_t_ means the concentration of PAH at time *t* during the UV irradiation, and *C*_0_ is the initial concentration of PAH. In addition, when the concentration of PAH is proportional to its fluorescence intensity, *C*_t_ and *C*_0_ could be replaced respectively by *F*_t_ (the fluorescence intensity of PAH at time *t*) and *F*_0_ (the initial fluorescence intensity of PAH) [30]. Many studies have shown that the photolysis of PAHs adsorbed on the plant leaves always follow first-order kinetics [14], [15]. Thus, the first-order reaction rates (*k*) and half lives (*t*_1/2_) for the photolysis of the PAHs could be obtained by the following equations [29]. 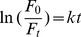
(1)

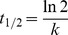
(2)


## Results and Discussion

### S-SSF spectra of An and Py adsorbed on the mangrove leaf surfaces

**Figure 2:**
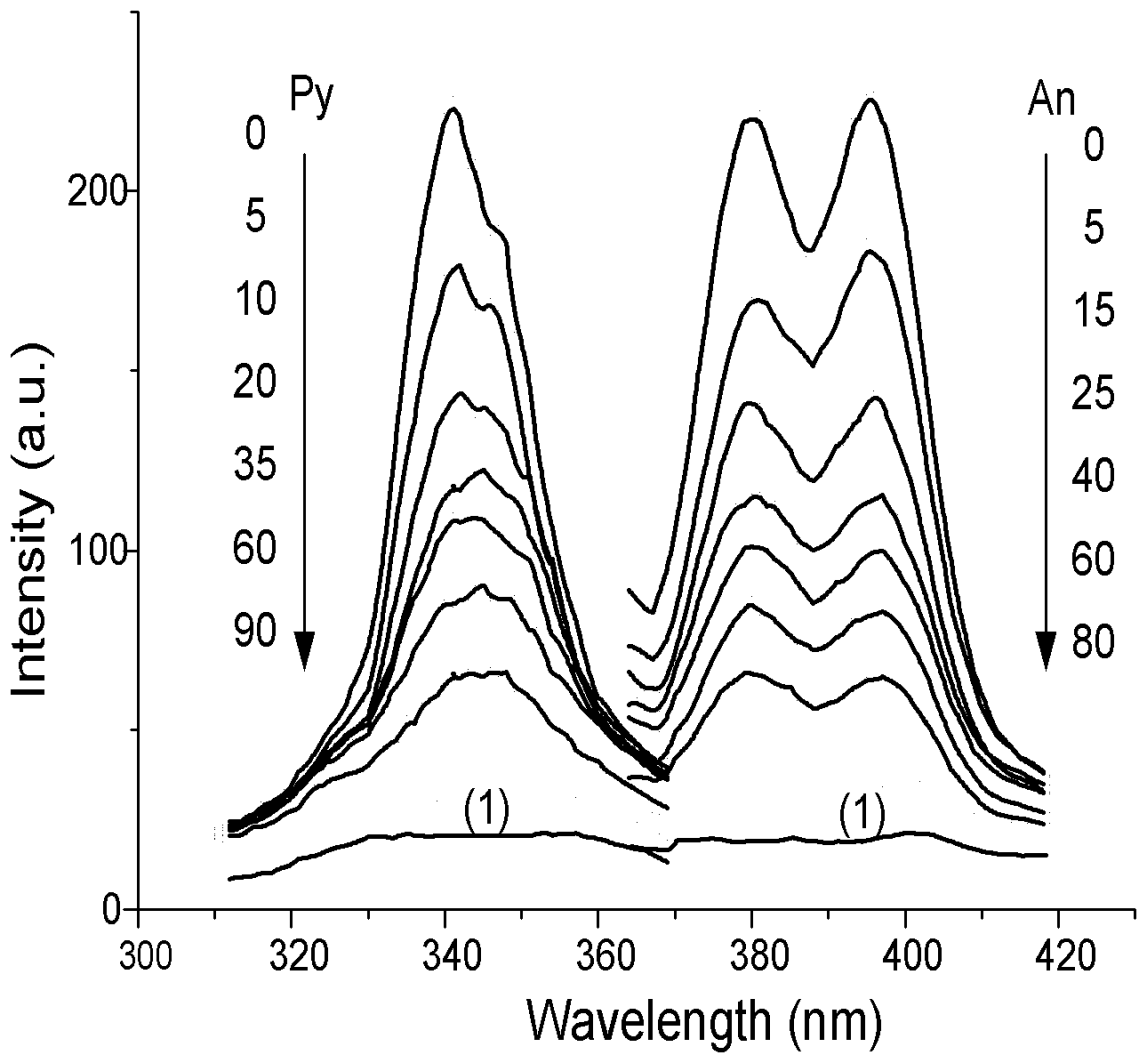
S-SSF spectra of the adsorbed An and Py on the *Ko* leaf surface. The order of the S-SSF intensity changes vs. time (min) is indicated by arrow. 1, uncontaminated *Ko* leaf.

In this work, the S-SSF established in our previous studies was utilized for *in situ* determination of An and Py adsorbed on the leaf surfaces of *Ko* and *Ac*, respectively [27]. Because the mangrove leaf is the complicated and heterogeneous media, the peak area spectrophotometry was not used for determination of the PAHs adsorbed on mangrove leaf surfaces. The wavelength of 387 nm and 343 nm was selected as the fluorescence spectra for the quantification of An and Py, respectively. It is reported that photolytic products of the PAH might be the main interference for *in situ* study of the fluorescence signals during photolysis [31]. Therefore, the S-SSF spectra of An and Py adsorbed on the leaf surfaces of two mangrove species should be investigated over time. Figure 2 showed the S-SSF spectra of An and Py adsorbed on the *Ko* leaf surfaces taken at different time during the irradiations. And the decrease of the relative S-SSF intensities was observed with time. As can be seen from Figure 2, the shape of the spectrum and width of the half-wide spectral band were no considerable differences over time. Thus, the photolytic products could not interfere with the detection of the An and Py adsorbed on the mangrove leaf surfaces. Moreover, the autofluorescence of the uncontaminated mangrove leaves was very weak at the selected spectrum, which could not affect the detection of the fluorescence signals either (Figure 2). There was also a similar trend of the photolysis of An and Py adsorbed on *Ac* leaf surfaces. Consequently, the S-SSF was acceptable to be utilized as an *in situ* method for the photolysis study of An and Py adsorbed on two specific mangrove leaf surfaces. Thus under laboratory conditions, photolysis might play a major role in the fate of PAHs adsorbed on mangrove leaf surfaces under UV irradiation.

### Study the photolysis processes of the An and Py adsorbed on the leaf surfaces of two mangrove species

**Figure 3:**
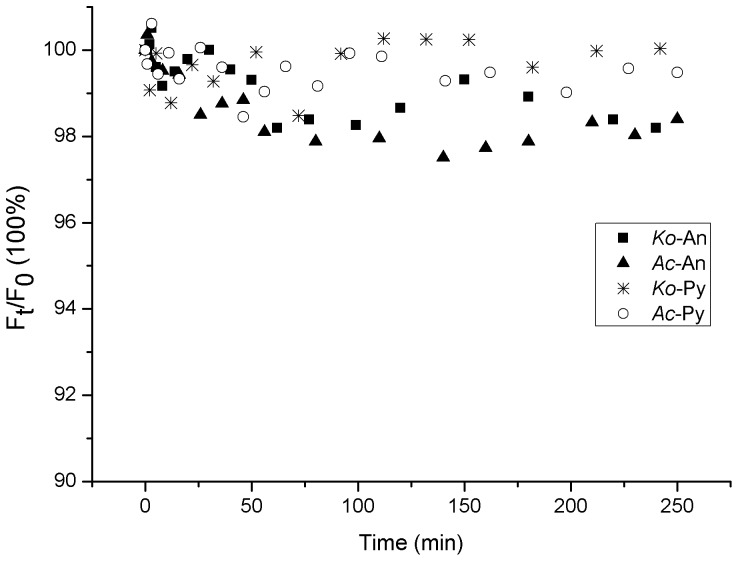
Variations of *F*_t_/*F*_0_ vs time of the adsorbed An (44.5 ng.spot^−1^) and Py (50.5 ng.spot^−1^) on the leaf surfaces of two mangrove species without illumination.

It has been shown that volatilization might be one of the significant pathways for PAHs loss from certain environment surfaces [32]. According to our previous studies, volatilization could be a major loss for the PAH adsorbed on leaf surfaces within six hours [33]. In this experiment, the illumination time was less than four hours. Thus, the decrease of fluorescence signals of the PAHs adsorbed on mangrove leaf surfaces might be primarily due to the volatilization from the leaf surface into the atmosphere, and the quantities of the PAHs entering into the inner leaf tissues could be neglected in a short-term experiment. In this work, the heating effects of mercury lamp whose light is guided by an optical fiber could be avoided. And results had shown that the temperature of the location placing the leaves was similar with room temperature. Thus, it is reasonable that the effects of evaporation for PAHs loss without illumination could be used as blank control. Therefore, the variations of *F*_t_/*F*_0_ without illumination were investigated over time during the photolysis processes of the PAHs adsorbed on the leaf surfaces of two mangrove species (Figure 3). The purpose of the non-irradiation control group is to investigate the effect of evaporation on the PAH disappearances. As can be deduced from Figure 3, the relative S-SSF intensities of the An and Py adsorbed on the leaf surfaces of two mangrove species decreased very little with the extension of time. Thus, the decrease of relative S-SSF intensities of the An and Py adsorbed on mangrove leaf surfaces might be all caused by photochemical reaction, and the quantities of An and Py volatilization from the leaf surfaces into the atmosphere could be negligible during a short-term experiment.

**Figure 4:**
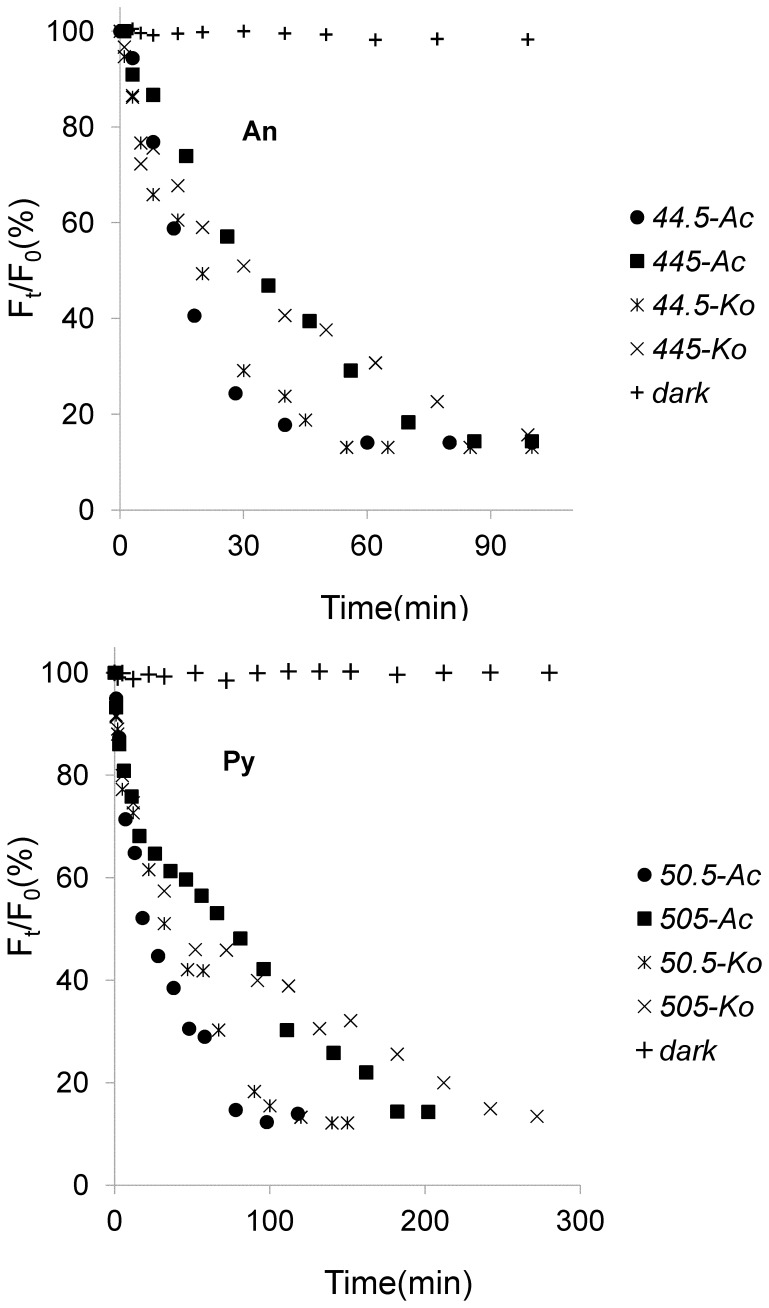
Photolysis processes of different initial amount of An (44.5 ng.spot^−1^, 445 ng.spot^−1^) and Py (50.5 ng.spot^−1^, 505 ng.spot^−**1**^) adsorbed on the leaf surfaces of two mangrove species.

It could also be deduced from Figure 3 that the decreasing extent of the relative S-SSF intensities of the adsorbed An was a little more than that of the adsorbed Py on the leaf surface of the same mangrove species with the extension of time. It is well known that the octanol-water partition coefficient (*K*_ow_) of the An is of lower value than the Py. More An might volatilize into the atmosphere under the identical experimental conditions [34]. In addition, for the same initial amount of PAH, the decrease of the relative S-SSF intensities of the PAH adsorbed on the *Ko* leaf surfaces was a little less than on *Ac* leaf surfaces (Figure 4). In this experiment, the leaf-wax content of *Ko* (7.63 mg.g^−1^) was much higher than the *Ac* (4.98 mg.g^−1^). Thus, the interactions between the adsorbed PAH and *Ko* leaf surfaces might be much stronger, which make the adsorbed PAH on *Ko* leaf surface difficult to volatilize [21], [27].

**Figure 5:**
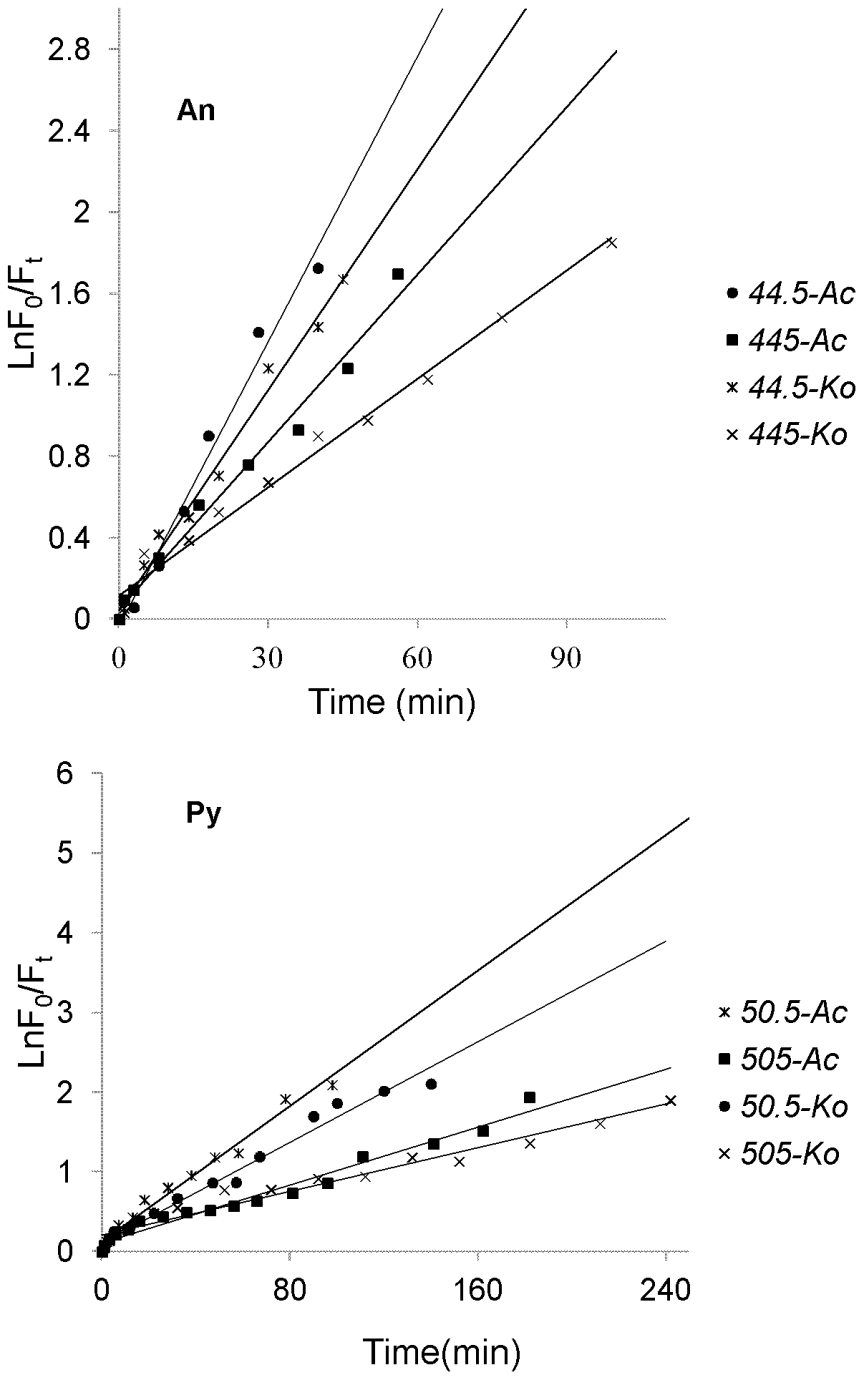
First-order kinetic plot of results of different initial amount of An (44.5 ng.spot^−1^, 445 ng.spot^−1^) and Py (50.5 ng.spot^−1^, 505 ng.spot^−1^) adsorbed on the leaf surfaces of two mangrove species (n = 9).

As mentioned above, the photolysis processes of An and Py adsorbed on the leaf surfaces of two mangrove species were firstly investigated in the course of time, and the experimental results were shown in Figure 4. As can be seen from Figure 4, the curves demonstrated the photolysis processes of each PAH were multi-decays. And within a certain period of time, the photolysis processes for both An and Py adsorbed on the leaf surfaces of two mangrove species followed first-order reaction kinetics with their photolysis rates in the order of *Ac*>*Ko* (Figure 5). Table 1 showed the kinetic results of the photolysis of An and Py adsorbed on the leaf surfaces of two mangrove species. It was shown from Table 1 that the photolysis half-lives of the lower initial amount of An (44.5 ng.spot^−1^) and Py (50.5 ng.spot^−1^) ranged from 14.8 min to 19.2 min and 32.5 min to 43.9 min, respectively. In addition, the photolysis half-lives of the higher initial amount of An (445 ng.spot^−1^) and Py (505 ng.spot^−1^) ranged from 25.3min to 38.9 min and 76.2 min to 101.9 min, respectively. Consequently, for both An and Py in the same experimental conditions, the higher the quantity of the PAH adsorbed on the mangrove leaf surfaces, the slower the photolysis rate of the PAH. Because each sample area on the leaf surface was fixed in the present study, the PAH adsorbed on the mangrove leaf surfaces might be of multi-layers, and the higher the initial amount of the adsorbed PAH, the thicker the PAH covered on the leaf surfaces [35]. Therefore, some excited states or polymer molecules of PAH might be formed on the leaf surfaces and absorb the photons of UV light. And the higher the quantity of the PAH adsorbed on the leaf surfaces, the more the excited states or polymer molecules of PAH formed. In conclusion, the number of photons striking the leaf surfaces decreased, and thereby decrease the photolysis rate of the PAH adsorbed on mangrove leaf surfaces [36]–[39]. However, the equations obtained in this work were not generalized. There are some limiting factors. Because of the multi-layers of the PAHs adsorbed on mangrove leaf surfaces, the UV photons accepted by the adsorbed PAHs on a different layer should be different, and the photolysis processes of the adsorbed PAHs should also be different. However, only a small amount of PAHs were selected in this work, the number of the layers could be small. Thus, the differences could be acceptable for *in situ* study the photolysis processes of PAHs adsorbed mangrove leaf surfaces in a short time. It was also from Table 1 that for the same amount of substances, the photolysis rate of Py adsorbed on the leaf surfaces of the same mangrove species was much lower than that of An. Thus, it was reasonable that the molecular structure might be one of the important factors for the photochemical behaviors of PAH [40], [41]

**Table 1:** Kinetic results of the photolysis of An and Py adsorbed on the leaf surfaces of two mangrove species (n = 9) and in water (n = 9).

Mangrove	PAHs (ng.spot^−1^)		Calibration carve	Correlation coefficient	*K*	*t* ^1/2^ (min)
*Ko*	An	44.5	*y* b = 3.62×10^−2^ *x* a+0.043	0.9952	3.62×10^−2^	19.2
		445	*y* = 1.78×10^−2^ *x*+0.115	0.9935	1.78×10^−2^	38.9
	Py	50.5	*y* = 1.58×10^−2^ *x*+0.114	0.9899	1.58×10^−2^	43.9
		505	*y* = 6.80×10^−3^ *x*+0.208	0.9802	6.80×10^−3^	101.9
*Ac*	An	44.5	*y* = 4.68×10^−2^ *x* - 0.036	0.9908	4.68×10^−2^	14.8
		445	*y* = 2.74×10^−2^ *x*+0.050	0.9926	2.74×10^−2^	25.3
	Py	50.5	*y* = 2.13×10^−2^ *x*+0.125	0.9865	2.13×10^−2^	32.5
		505	*y* = 9.10×10^−3^ *x*+0.109	0.9898	9.10×10^−3^	76.2
water	An	44.5	*y* = 5.22×10^−2^ *x*+0.042	0.9960	5.22×10^−2^	13.3
	Py	50.5	*y* = 2.88×10^−2^ *x*+0.088	0.9937	2.88×10^−2^	24.1

^a^ stands for the illumination time of the PAH adsorbed on mangrove leaf surface.

^b^ stands for the value of ln(*F*_0_/*F*_t_).

It is supposed that the photolysis of PAHs on plants predominantly take place in the cuticle and especially the cuticular wax coating on the leaf surfaces [19]. Previous studies found that the wax or lipid on the plant leaves could absorb the UV photons, which might create the light filtering effect. Thus the number of the UV photons striking the PAH adsorbed on mangrove leaf surfaces might decrease, and thereby decrease its photolysis rate [14], [15]. Because the leaf-wax content of *Ko* (7.63 mg.g^−1^) was much higher than the *Ac* (4.98 mg.g^−1^) in this experiment, the light filtering effects of *Ko* leaves might be stronger than *Ac* leaves. Consequently, the photolysis rate of the same initial amount of PAH adsorbed on *Ko* leaf surfaces was much slower than that adsorbed on *Ac* leaf surfaces (Table 1). In addition, the relative S-SSF intensities of the each PAH had little change when the amount of each PAH degraded to about 12% of the initial amount (Figure 4). In other words, the photolysis processes of the residual PAH adsorbed on the mangrove leaf surfaces could not occur with the illumination time prolonging. Xu et al and Schuler et al also found the same phenomena in their studies. However, they did not give the possible reasons [42], [43]. Combined with others and our present studies, a little amount of PAH might enter into the inner leaf tissues and could not absorb the UV photons because of the light filtering effects of mangrove leaf surfaces. On the other hand, the generated photolytic products of the PAH in the upper layers might cover on the residual PAH in the low layers during illumination. Thus, the photolysis rates of the PAHs in the low layers might decrease. As time goes on, the photolysis of the residual PAH in the low layers might not occur [14], [15]. Certainly, further studies are also needed.

### Comparisons of the photolysis of An and Py adsorbed on mangrove leaf surfaces and in water

**Figure 6:**
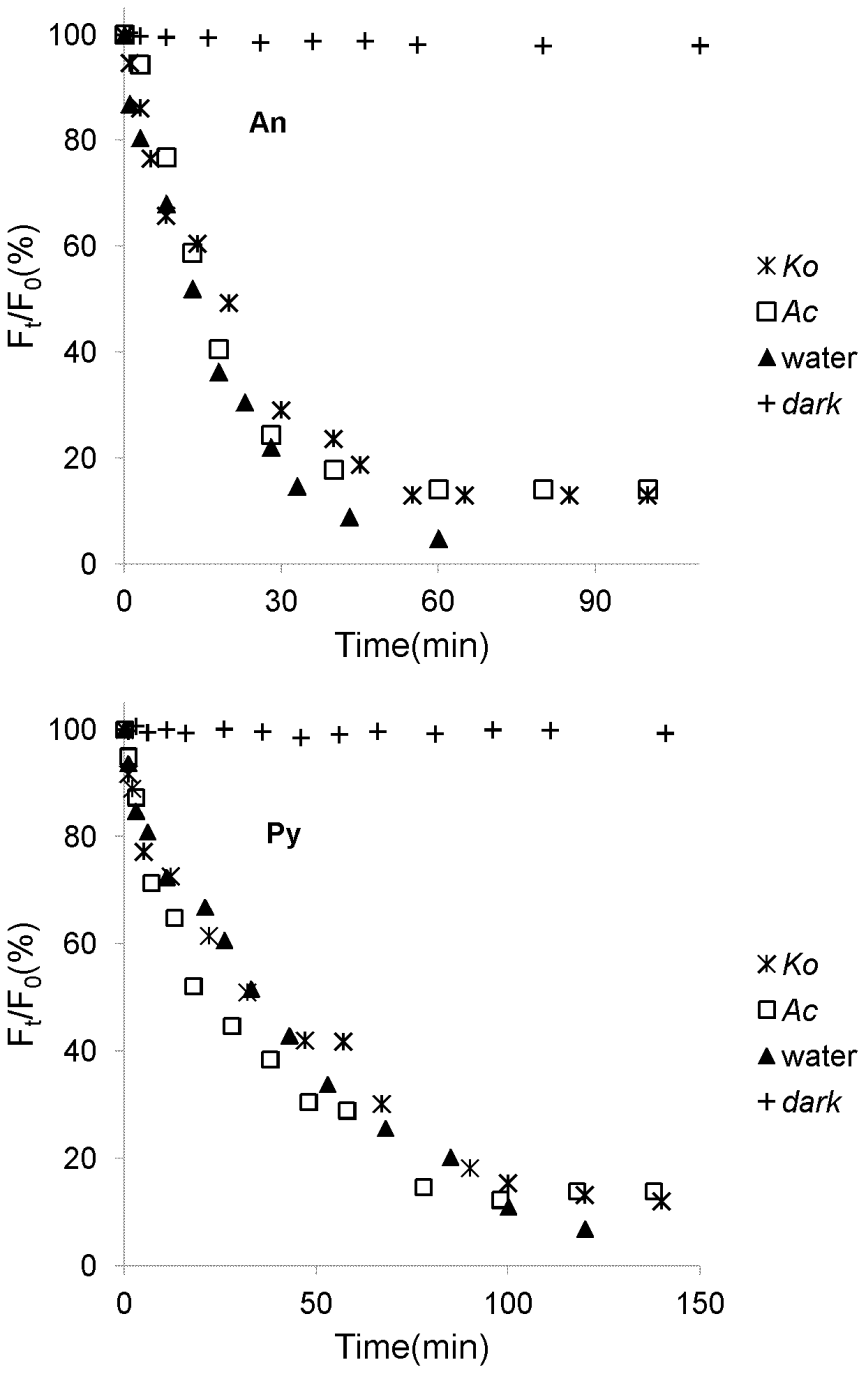
Photolysis Processes of the same initial amount of the An (44.5 ng.spot^−1^) and Py (50.5 ng.spot^−1^) adsorbed on two typical mangrove leaf surfaces and in water.

**Figure 7:**
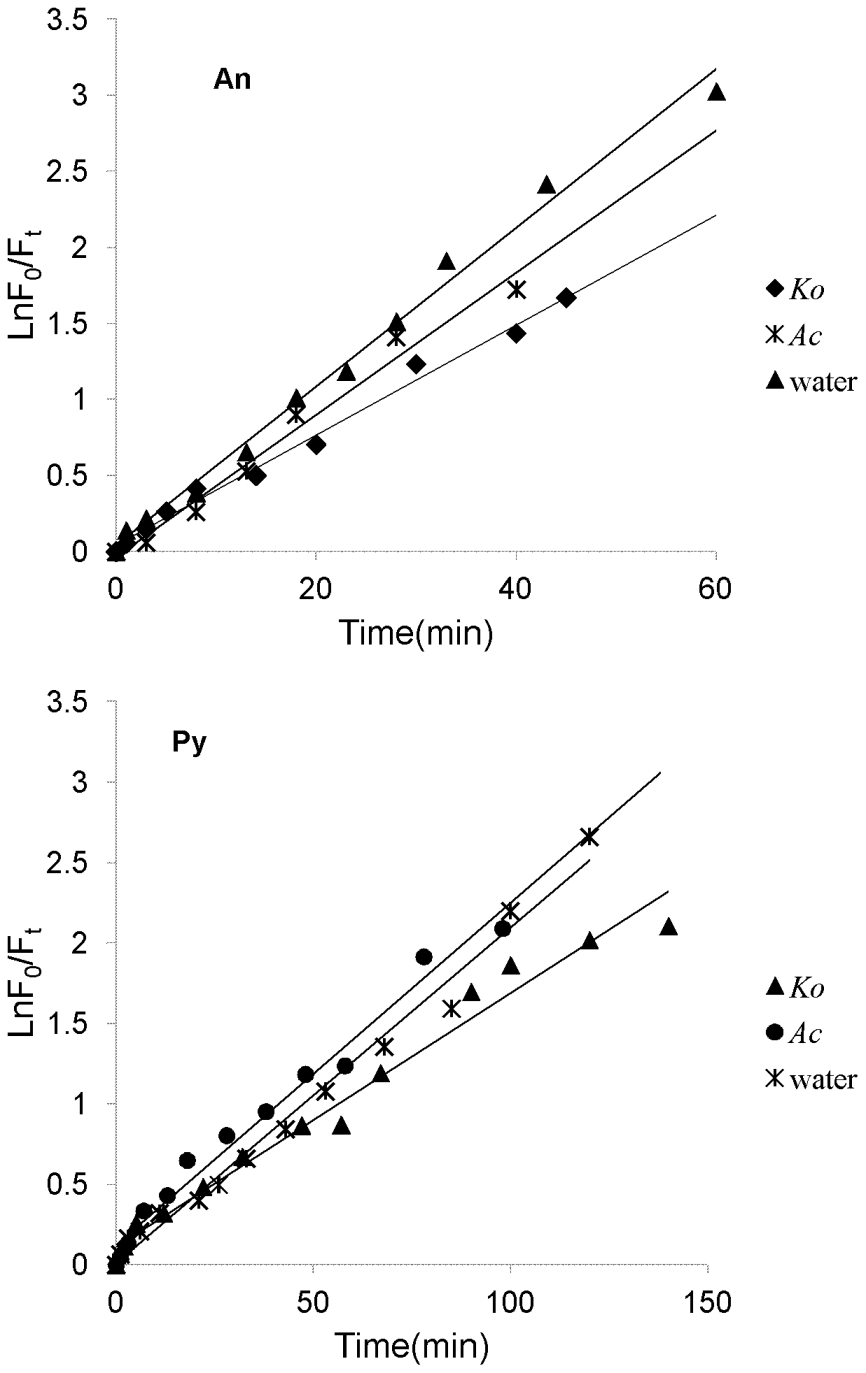
Variation of *F_t_/F_0_* vs time during photolysis of the same initial amount of the An (44.5 ng.spot^−1^) and Py (50.5 ng.spot^−1^) adsorbed on two typical mangrove leaf surfaces and in water.

According to previous reports, the more polar the solvent is, the faster the photolysis processes of PAH [43], [44]. Thus, the photolysis rate of PAH appears to be highly dependent on the substrate on which they are adsorbed. In order to further study the possible photolysis mechanisms of the PAHs adsorbed on mangrove leaf surfaces, the photolysis processes of An (44.5 ng, 0.25 nmol) and Py (50.5 ng, 0.25 nmol) in water were also investigated for comparison (Figure 6). The fluorescence method for determination of the An and Py in water has been established in our previous studies [29]. As can be seen from Figure 6, the PAH in water could be degraded continually without stop under the same light intensity. It was different with the photolysis processes of the PAH adsorbed on mangrove leaf surfaces. Figure 7 showed the variations of *F*_t_/*F*_0_ vs time during the photolysis processes of An and Py adsorbed on the leaf surfaces of two mangrove species and in water. From the Figure 7, it was evident that the photolysis of An and Py dissolved in water also followed first-order reaction kinetics, respectively. With same amount of substances, the kinetic results of the photolysis of An and Py adsorbed on the leaf surfaces of two mangrove species and in water were shown in Table 1. According to the data of Table 1, the photolysis half-lives for An adsorbed on *Ko*, *Ac* leaf surfaces and in water were 19.2, 14.8 and 13.3 min, respectively, while the photolysis half-lives for Py adsorbed on *Ko*, *Ac* leaf surfaces and in water were 43.9, 32.5 and 24.1 min, respectively. These results indicated that the photolysis rate of the same PAH was much faster in water than on mangrove leaf surfaces. Mangrove leaf surfaces are covered by thick cuticle consisting of many long chain saturated and unsaturated fatty acids [18]. Consequently the polarity of mangrove leaf surfaces might be weaker than that of water. Thus, it was reasonable that the photolysis rate of the same PAH adsorbed on mangrove leaf surfaces was slower than in water. Moreover, some of the lipoidal compounds on plant leaves could adsorb and reflect the UV light [26], [27], [36]–[38]. And the number of UV photons striking to the PAH adsorbed on the mangrove leaf surfaces decreased, and thereby slowed down the photolysis rate of the adsorbed PAH. For this reason, the photolysis mechanism of the same PAH adsorbed on mangrove leaf surfaces and in water might be different, further studies are therefore needed.

## Conclusions

Using the established S-SSF method, the photolysis processes of An and Py adsorbed on the leaf surfaces of two mangrove species were *in situ* studied for the first time. Meanwhile, the photolysis for both An and Py adsorbed on the leaf surfaces of two mangrove species followed first-order reaction kinetics with their photolysis rates in the order of *Ac*>*Ko*. In addition, the higher the quantity of the PAH adsorbed on the mangrove leaf surfaces, the slower the photolysis rate of the PAH. Moreover, for the same amount of substances, the photolysis rate of Py adsorbed on the same mangrove leaf surfaces was much lower than that of the An, and the photolysis rate of the same PAH in water was much faster than on mangrove leaf surfaces. Thus, photochemical behaviors of PAHs were dependent not only on their molecular structures but also the physical-chemical properties of the substrates on which they are adsorbed.

The S-SSF method is simple, accurate and easy operating for *in situ* investigation the photolysis of the PAHs adsorbed on mangrove leaf surfaces. However, much more studied need to be carried out in the near future. For example, PAHs usually exist in a multi-component in real environment, and this S-SSF might be a good way to be used for determination of multi-component PAHs simultaneously. It could be prospected that with the development of the S-SSF methods, it would be helpful for us to understand more the environmental behavior of PAHs in real environment, and it would also provide us a new way to study on mechanisms of phytoremediation of PAHs in mangrove wetland or other contaminated mediums.
